# Anesthesia Provision in Disasters and Armed Conflicts

**DOI:** 10.1007/s40140-017-0190-0

**Published:** 2017-02-16

**Authors:** Miguel Trelles Centurion, Rafael Van Den Bergh, Henry Gray

**Affiliations:** 1 Surgical Care Unit – Operational Centre Brussels, Médecins Sans Frontières – Doctors Without Borders, Rue de l’Arbre Bénit 46, 1050 Brussels, Belgium; 2 Operational Research Unit – Operational Centre Brussels, Médecins Sans Frontières – Doctors Without Borders, Rue de l’Arbre Bénit 46, 1050 Brussels, Belgium; 3 Emergency Unit – Operational Centre Brussels, Médecins Sans Frontières – Doctors Without Borders, Rue de l’Arbre Bénit 46, 1050 Brussels, Belgium

**Keywords:** Anesthesia, Surgery, Disasters, Armed conflict, Ketamine, Precarious settings

## Abstract

**Purpose of review:**

Disasters and armed conflicts are characterized by high numbers of trauma cases, and occur mainly in developing countries where the healthcare response is already impaired, resulting in an inadequate response. Aside of the trauma cases, other surgical health conditions are also still present and require urgent care. Surgical care needs are different from context to context and depend on local means and capabilities.

**Recent findings:**

Doctors without Borders (MSF) has proven that even in precarious situations, safe administration of anesthesia is possible, and the “do no harm” principle can and must be upheld. Anesthesia providers need to recognize the difficulties linked to these contexts.

**Summary:**

Local, spinal and general intravenous (mainly with Ketamine) anesthetics seem to be the most widely accepted. Inhalation anesthesia has constraints; regional is underused and epidural is not recommended. Standard operative procedures should be in place, and an informed consent from the patient must be granted.

## Introduction

Situations of disaster and armed conflict are typically linked with high numbers of trauma cases, in contexts where the healthcare response is compromised through damaged infrastructure and scarcity of human resources. Additionally, disasters and armed conflicts are more likely to occur in developing countries where healthcare systems are often already lacking material and human resources. These conditions typically result in a weak healthcare response to the sudden event (in case of disasters) or to the sudden event that then evolves into a chronic situation (as is often the case for armed conflicts). These situations not only affect the care for the (usually trauma) cases resulting directly from the event, but also compromise the care for health issues that are still present in the affected population and that require immediate care, such as emergent obstetric care.

Healthcare delivery in general differs considerably between disasters and armed conflicts, and between different types of armed conflicts, in terms of scale, who is affected, anticipated morbidity patterns, and contextual limitations. These patterns have also changed over time. In the early twenty-first century, major armed conflicts between opposing countries have been few (e.g. the 2008 Russian – Georgia armed conflict), and did not result in large-scale attacks against the civilian population. Instead, conflicts have principally been internal, with or without the involvement of international forces (e.g. respectively Afghanistan and the Chechen conflict in Russia). A relatively new characteristic, further compromising healthcare delivery, is the lack of respect for the sanctity of healthcare structures and the staff working in them (with notable incidents in Kunduz in Afghanistan, Aleppo in Syria, and Saada in Yemen): if in previous armed conflicts the healthcare system was mainly affected due to the absence of human resources and difficult supply lines, nowadays it is more and more a target by itself, and suffers the destruction of facilities and killing of staff [1, 2]. Another important differentiation in healthcare delivery is the difference between the military and civilian healthcare response. The military mainly takes care of own combatants who were screened for their health status before combat and are generally healthy and young; facilities are typically well-resourced; and patients in severe conditions usually have the possibility for referral out of the conflict zone for further treatment and rehabilitation. Civilian healthcare structures in conflict zones do not have these characteristics.

The provision of surgical care in such settings has been described extensively in the literature, but the determinants of anesthesia provision in settings of disaster and/or armed conflict have not been documented in the same detail. While the required anesthesia techniques as such may be the same for both disasters and armed conflicts, it is important to frame these needs against the availability of local resources in place (infrastructure, material, equipment and human resources), the possibility and availability of supply lines, and the magnitude of casualty numbers and other health needs. Even if the conditions are very difficult and unfavorable during disasters and armed conflicts, safe administration of anesthesia remains possible and should be provided with the best possible quality that can be offered in a specific context. The “*do no harm*” principle must regulate the anesthesia provision and has to be respected at all times [3••].

Médecins Sans Frontières (MSF, also known as Doctors without Borders) is a humanitarian medical non-governmental and non-profit organization that has been providing surgical care for more than 45 years in disasters and armed conflicts, mainly in resource-poor settings. It follows its institutional charter: assistance to populations in need, irrespective of race, religion, creed or political convictions. MSF actions are guided by medical ethics and the principles of independence, neutrality and impartiality. MSF has an important experience in the provision of surgical care, including safe anesthesia, in highly challenging settings – this experience principally relies on a wide flexibility, and delivery of relatively standardized care, which is only minimally tailored to a given setting; and also, for which standard operative procedures have been developed [4].

## Resource Requirements and the Aid Response

In developing countries, qualified surgical and anesthesia providers are typically rare to non-existent, and this shortage is also mirrored in a lack of minimal infrastructure and supplies to perform surgical activities. In general, if availability of surgical providers is already a concern, the availability of anesthesia providers is far more limited, in turn restricting the possibilities of surgical care and in some conditions even making it dangerous. This is a common reality in resource-poor settings, though recent reports suggest an improvement of the conditions for the provision of surgical care in several developing countries [5•].

In disasters, surgical care needs differ from context to context and mainly depend on the existing local means and capabilities [6]. A disaster event with high numbers of casualties places the existing healthcare system under high pressure, and in most cases will require external resources (national or international) at least during the first days following the disaster, as health needs overwhelm the local capacity during the immediate aftermath [7]. Unfortunately, relief aid cannot be provided immediately and the affected population will initially rely on the remaining healthcare providers in the area [8]. This underscores the importance of deploying external resources with the shortest delay: to take care of patients in need that do not have access to healthcare, and to support the local healthcare providers who will rapidly be exhausted. An important determinant of the type of post-disaster support is the type of setting where the disasters occurs, e.g. in a major city (such as the 2010 Haiti earthquake that hit the capital, Port-au-Prince) or in rural areas without affecting major cities (such as the 2015 Nepal earthquake, which left the hospitals in the capital, Kathmandu, relatively untouched). In the first case, relief aid focused on replacing the destroyed health infrastructure, with specialized international human resources providing important and unprecedented aid; while in the second case, aid focused on non-specialist care, mainly consisting of rescue and transport of patients to existing health facilities. Nevertheless, in Nepal specialized human resources were also deployed by different relief organizations, in order to help the local healthcare system cope with the high number of casualties.

In such contexts where provision of surgical care is difficult, it is not recommended to extensively substitute the local workforce with international or national staff shifted from other health facilities. If the new external staff (international or national shifted from elsewhere) works alone, there will be difficulties in understanding the local traditions and habits, as well as local social and ethical norms. At the same time, local staff may move away as no work possibilities are available, which can considerably challenge the handover when the external staff tries to leave the affected region in a responsible way. A balance should be found, promoting collaboration and sharing with locally embedded individuals, to build towards a handover of activities within a defined time [9]. To this end, anesthesia techniques should answer to available local skills and knowledge. In all cases, sustainability after the exit of external aid should be assured and planned in advance where possible: using drugs and material available in the local context, and implementing techniques that could be easily followed without endangering patients.

In terms of material supplies, challenges to reliable supply chains in disasters and conflicts encompass practical issues such as distance from suppliers to the end users; safety of the supply routes; ensuring cold chain conditions in areas where shipments may be held for weeks; availability of temporary stocks; and waiting for access to improve. Additionally, required quantities of supplies can be challenging to assess – storage capacity may be limited, and re-supply timing unpredictable. An understanding of the epidemiological context, both before and during the disaster or armed conflict, is essential to avoid shortages in specific supplies [10]. An additional challenge related in particular to provision of anesthesia in disaster- and/or conflict-affected settings is importation of controlled substances/narcotics into third-party countries and subsequent exportation to contexts where importation procedures are dysfunctional. A salient example is the regulatory framework concerning Ketamine, which is considered a narcotic in some countries (such as Belgium, from where a major MSF section operates) but not in others. For MSF, export of Ketamine under non-emergency conditions requires an import permit from the local authorities where the product will be used; transmission of the import permit to Belgian authorities for approval and issuing of an export permit; packing of the medication; and finally verification and sealing of the order by the Belgian authorities. Under emergency conditions, or if no functioning local authority is available, the import permit can be waived, but is replaced by an emergency letter from the medical director of the organization, which must stipulate the precise quantity, formulation and dosage of the medication. The slightest error against any of these details compromises the shipment and can result in delays of weeks or months.

## Constraints to Anesthesia Provision

While surgical management and the required competencies for providing surgery in disaster and armed conflict situations have been widely studied, anesthesia management remains an underreported activity. This is a concern, as anesthesia management is an important component of surgical care. With the exception of reports by the military, most published information is generated by anesthesia providers of humanitarian organizations (international aid) or emergency teams (national aid) that supported surgical care activities in disaster and/or armed conflict settings. Here, we do not address the anesthesia care provided by military actors, as the resources at their disposal are much more vast than those for civilian providers.

In order to adequately plan quality anesthesia management, civilian anesthesia providers need to recognize the constraints that are linked to the precarious settings in which they work [11]:

- *Healthcare system characteristics*: major constraints can occur at the level of the facility in which surgical care is to be provided [12]. When hospitals are no longer functional, other structures such as houses or schools can be converted to provisional hospitals. Such atypical settings have a large impact on healthcare provision, including difficulties in assuring basic levels of asepsis and antisepsis for surgical interventions (lack of an adequate room, of sterilization facilities and even of surgical material); scarce human resources (not only qualified personnel) as people might have abandoned the location; and limited or inexistent diagnostic tools and therapeutic means. Referrals may not be possible, and the surgical team can end up being the last resource of patients.
- *Triage characteristics*: As surgical needs overwhelm the surgical capacities, a good triage should be implemented quickly. Triage in disasters and armed conflicts differs considerably from triage used in routine settings: the main goal in disasters / armed conflicts is to provide the best possible surgical care to the highest number of casualties, usually with limited resources, while triage in routine settings is aimed at establishing a priority between patients but doing all that is possible for every patient. When working in such contexts, it is important to accept the limits inherent to surgical care, while always upholding the principles of medical ethics.
- *Patient characteristics*: Typically, in addition to the high volume of trauma casualties, often exceeding the healthcare system’s capacity, surgical needs for non-trauma pathologies are also present, with emergent obstetrical interventions remaining one of the main surgical procedures conducted in these situations [13]. Additionally, communicable and non-communicable diseases may be present as co-morbidities among surgical patients, as the sudden event typically hits the local population without any type of differentiation. Other challenges to provision of quality care can include: late arrival at the health facility due to poor accessibility (destroyed or inexistent roads, lack of transportation means, security issues), aggravating the health status of the patient; lack of previous medical records, as patients may arrive from different locations or may not have had good access before the event; unique patterns of wounds, which may differ from common accidental trauma [14, 15]; or urban violence with multiple injuries; patients might be dehydrated; and crush syndrome may occur in certain types of disasters, such as earthquakes with many individuals trapped under rubble for extended periods of time [16].
- *Anesthesia management characteristics*: As health infrastructure may be compromised or inexistent, the standards can be very basic. A general lack of equipment for patient follow-up may exist: electronic monitoring may not be present or the electricity supply may be unreliable, forcing the anesthesia provider to manually monitor the patient, and oxygen provision may be difficult and in some cases even impossible. There can also be difficulties performing a correct pre-operative assessment due to lack of medical registers or to the high workload; difficulties to perform intubated anesthesia as ventilators might not be present; unavailability of an assistant; and difficulties to offer a proper and closed observation of the patients while they recover from the anesthesia.

The experience of MSF in the aftermath of the January 10th, 2010 Haiti earthquake is valuable to understand the type of anesthesia techniques that are commonly used in disasters. In that exceptional situation, MSF deployed an unprecedented number of emergency teams, which included emergency medicine physicians and nurses, surgeons and orthopedic surgeons, anesthetists, and theater nurses, in an effort to provide quality surgical care to the population in distress. One of the health structures where MSF deployed its teams was in the neighborhood of Cité-Soleil in Port-au-Prince in the “Centre Hospitalier Sainte Catherine Laboure”, commonly known by the local population as Choscal. Surgical activities could only be performed from January 16th onward, as several conditions needed to be met: securing the hospital infrastructure to resist the numerous aftershocks; organization of the staff of the hospital who were scattered with their family all around the city without communication means; bringing the required supplies to the country; and assuring the presence of international specialized staff. During the first 44 days of intensive work, 325 patients (new cases) were operated upon, of whom 60% were accidental trauma mainly related to the earthquake. Among these patients, 678 surgical interventions (all cases) were performed, of which 72% were minor/wound care. The higher number of surgical interventions is linked to the need for several re-interventions; a characteristic of trauma care. The provided anesthesia care included: general non-intubated for 66% of anesthesia’s, general intubated for 9%, spinal for 21% and other anesthetics for 4%. Detailed information is provided in Table 1.

**Table 1:** Activities performed in Choscal, from January 16th to February 28th, weekly

Epidemiological Weeks	**2**	**3**	**4**	**5**	**6**	**7**	**8**	**Total**
Days	Jan 16–17	Jan 18–24	Jan 25–31	Feb 1–7	Feb 8–14	Feb 15–21	Feb 22–28	
**Causes of intervention**	**14**	**71**	**44**	**37**	**51**	**54**	**54**	**325**
– Accidental Trauma (60%)	6	63	37	18	31	26	13	194
– Violent Trauma (18%)	1	5	6	7	10	9	17	55
– Non-Trauma (11%)	0	1	1	3	5	16	12	38
– Maternal (11%)	7	2	0	9	5	3	12	38
**Type of intervention**	**15**	**102**	**123**	**121**	**118**	**111**	**88**	**678**
– Obstetrics (6%)	7	2		9	5	3	13	39
– Visceral (7%)	4	7	4	7	2	13	8	45
– Orthopedics (15%)	3	37	27	8	11	14	5	105
– Minor / wound (72%)	1	56	92	97	100	81	62	489
**Type of anesthetics**	**15**	**102**	**123**	**121**	**118**	**111**	**88**	**678**
– General (66%)	4	31	85	92	98	85	53	448
– Intubated general (9%)	3	15	8	10	2	11	10	59
– Spinal (21%)	8	50	24	18	13	11	20	144
– Others (4%)	0	6	6	1	5	4	5	27
**Order of intervention for earthquake related accidental trauma surgery**	**7**	**93**	**116**	**101**	**87**	**70**	**40**	**514**
– First intervention (37%)	6	63	37	18	30	22	12	188
– Re-interventions (63%)	1	30	79	83	57	48	28	326

When considering armed conflicts, two types of context should be differentiated. The first is when there is an eruption of violence that lasts some weeks but quickly sees an end to hostilities; a context we could term an acute armed conflict (e.g. the 2011 Côte d’Ivoire turmoil). The second, termed a chronic armed conflict, is when after an eruption of violence there is no quick end of hostilities, but rather they are protracted over months or years (e.g. the ongoing Syrian civil war).

**Fig. 1:**
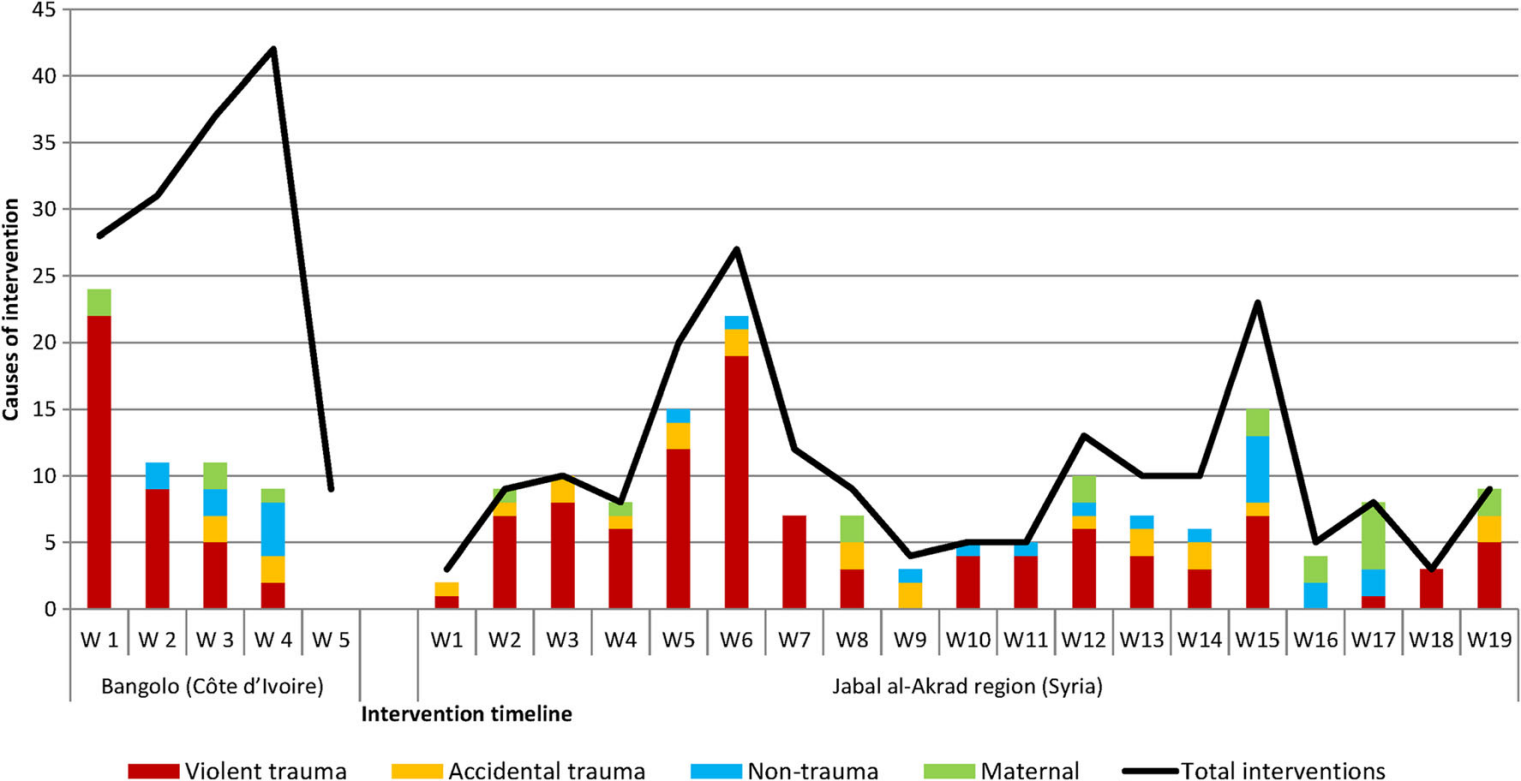
Causes of intervention (new cases) and surgical interventions (all cases) in MSF structures in Bangolo (Côte d’Ivoire) and in the Jabal al-Akrad region (Syria), from the start of the MSF activities in the region

During the 2011 Côte d’Ivoire turmoil, MSF ran a short intervention nearby Bangolo, in the west of the country, around 250 km from the capital Yamoussoukro. Surgical care was provided to 55 patients, of whom 69% suffered from trauma related to violence. 147 surgical interventions were performed, of which 78% were minor/wound care. The performed anesthesia techniques were in the majority general non-intubated (71%), and also general intubated (12%), spinal (8%), and other anesthetics (9%). One of the characteristics of surgical care provision in this context is that at the beginning of the intervention there is a high caseload (new cases) that gradually decreases, while the workload (all cases) gradually increases due to the cumulative effect of re-interventions for trauma care (Figure 1).

Before security constraints prohibited the activities of surgical care teams with international staff in Syria, MSF provided surgical care in provisional adapted structures in the north west of the country in the Jabal al-Akrad region from September 2012 to December 2013. In this setting, surgical care was provided to 578 patients, of whom 44% suffered a trauma due to violence. 712 surgical interventions were performed, of which 51% were minor/wound surgery. The performed anesthesia techniques were again in majority general non-intubated (48%), and also general intubated (18%), spinal (28%), and other anesthetics (6%). The main difference in this setting, compared to acute armed conflict context, is that surgical care provision should continuously be prepared for peaks in activities due to a high afflux of casualties after a clash or to the arrival of all kind of patients if suddenly accessibility is granted (Figure 1). This irregular daily caseload places considerable pressure on the provision of care.

## Anesthesia Techniques in Disaster and Armed Conflict Settings

In precarious contexts, some anesthesia techniques may not be appropriate. The guiding principles are that they should take into consideration two main features: as much as possible, cardio-respiratory depression and muscular relaxation should be avoided, and limited dependence on oxygen and biomedical monitoring should be maintained [17].

The following techniques appear to be widely accepted:

- *Local anesthesia*: It is considered a good technique for many indications as it is safe and effective. As some risks are present (e.g. cardiac toxicity after accidental intra-venous injection), it should be performed in defined areas (e.g. emergency or operating departments) where material and equipment for resuscitation maneuvers are present.
- *Spinal anesthesia*: This technique, if correctly performed, is also safe and effective, and oxygen availability is not an absolute requirement. It is recommended in surgical interventions under the umbilicus: lower limb surgery, obstetrics, and surgery in the pelvis and inguinal area. Nevertheless, as any anesthesia, means should be available for timely resuscitation and to overcome possible adverse effects (e.g. hypotension).
- *General intravenous anesthesia*: This technique can be performed with and without muscular relaxation. In precarious situations, the decision of neuromuscular relaxation should be taken with care, in function of the availability of mechanical ventilation. In some cases it will be necessary to keep patients in spontaneous ventilation during long periods of the intervention, as manual ventilation limits the activities of the anesthesia provider [18].

Ketamine remains one of the preferred options for intravenous anesthesia in precarious situations [19, 20, 21, 22, 23]: it has analgesic and narcosis effects that avoid the use of opioids during the surgical intervention; it can be used for almost all types of surgery; and as laryngeal reflexes are not totally suppressed, it allows performing some interventions without intubation (e.g. where anesthesia providers are not highly skilled, general intravenous anesthesia with Ketamine and without intubation is used for Cesarean sections). It should be acknowledged that the main concern in disasters and armed conflicts is hemorrhagic shock (from trauma, obstetrical or visceral origin), and the effects of Ketamine in the cardiovascular and respiratory systems can only be positive when dealing with this particular killer.

Some anesthesia techniques have been reported by some emergency teams in precarious settings, though implementation is debatable:

- *Inhalational anesthesia*: This type of technique might not be possible to implement as serious concerns might be present: lack of anesthesia circuits, difficulties in supply of halogenated anesthetics, challenges to properly evacuate these anesthetic agents from improvised operating rooms. Its implementation will also demand the availability of narcotic drugs for analgesia [24]. Good solutions for replacing mechanical devices are available, where draw-over manual anesthesia circuits can be a good option: they can be transported by the anesthesia provider itself.
- *Regional anesthesia*: This is a technique that unfortunately is underused in many settings, despite its positive characteristics in surgical management of limb trauma. It is safe and efficient, and does not rely on the availability of oxygen. Its implementation is more linked to the knowledge and skills of the anesthesia provider, and to a timely availability of not only specific needles and anesthetics, but also of specific devices such as nerve stimulators or ultrasound. Several reports propose more use of this technique in precarious settings, and emergency teams should think on how improve its provision [25, 26, 27].
- *Epidural anesthesia*: In precarious settings, the implementation of this technique might be difficult and may not be recommended. The possible complications outweigh its possible benefits, as in these settings it is always possible to use spinal technique. Long lasting analgesia with the help of epidural catheters can be difficult to achieve and complications such as hematomas or headaches can be a burden for anesthesia providers that are already overloaded of work. However, in more controlled settings with better resources, this technique could be implemented [28].

It has been documented that the use of standard operative procedures provides coherence and good understanding to the management and treatment of patients, and certainly improves the quality of the provided healthcare. The standardization of care given by different anesthesia providers eases the work of the pharmacy and supply, and avoids confusion among the healthcare staff. The implementation of standard operative procedures should be one of the first activities to be performed in difficult settings; the most important components are: antibiotic prophylaxis and treatment, post-operative pain management, thromboprophylaxis, blood transfusion, and monitoring of surgical site infections.

Lastly, though not less important, surgical procedures must only be performed if there an informed consent has been granted by the patient, or if incapable, by her/his representative. This is highly recommended by the WHO, in order to preserve the patient’s autonomy and her/his right to even refuse the surgical intervention. In precarious contexts when life-threatening situations need urgent care, the surgical team can be confronted with the impossibility of obtaining the informed consent from the patient. In such situations, the case must be exhaustively documented and medically justified for the patient’s best interest.

## Conclusions

Quality anesthesia provision in disasters and armed conflicts remains a challenge, due to among others impaired healthcare systems, security concerns, challenges in ensuring supply lines, and lack of adequate human resources and material. However, following the experience of several surgical care teams, it is possible to perform safe anesthesia in such contexts, by carefully considering the need for a satisfactory infrastructure, basic means (disposables, drugs, biomedical devices), competent human resources, standard operative procedures, and adapted anesthesia techniques. The provision of safe anesthesia should be tailored to a specific context, aiming for the best possible quality that can be offered. The “*do no harm*” principle must be upheld at all times and surgical care should not be provided at any cost, compromising safety and quality.

Local, spinal and general intravenous are the recommended techniques in precarious situations. Ketamine, as an intravenous agent, remains the drug of choice in such contexts due to its pharmacological characteristics, though issues with importation and exportation need to be taken into consideration. Inhalational anesthesia might not be possible to implement, while regional anesthesia is an underused technique that should be encouraged in the future. Epidural anesthesia is not recommended in these settings as it is possible to perform spinal anesthesia and avoid potential complications.

Clear standard operative procedures should be implemented in these contexts to ease the provision of care and improve its quality. At the same time, clear policies regarding informed consent form should be in place as it is a right of the patient to know what kind of procedure is going to be performed and what the possible risks are, and to be able to refuse the proposed surgical care.

There are few reports regarding anesthesia management in disasters and armed conflicts, mainly written by relief teams that worked for a short period in these contexts. It is important to encourage local anesthesia teams and researches to report their invaluable experience working in precarious situations. Sharing experience will help other anesthesia providers working in similar situations and international / national aid organizations to deliver safe and quality anesthesia in ongoing and future situations of disaster and armed conflicts.
